# Supplementary material for: Metabolic Biomarkers of Liver Failure in Cell Models and Patient Sera: Toward Liver Damage Evaluation In Vitro
Source: Int J Mol Sci. 2024 Dec 23;25(24):13739. doi: 10.3390/ijms252413739 (PMC11677895; doi:10.3390/ijms252413739)
Supplement: Supplementary file 1 [file ijms-25-13739-s001.zip › ijms-3318503-supplementary.pdf]

Supplementary Information: Metabolic biomarkers of liver failure in cell models and patient sera: towards liver damage evaluation *in vitro*

Supplementary Table S1. Characteristics of PHH donors obtained from PRIMACYT Cell Culture Technology GmbH, Schwerin, Germany.

	HH180109	HH180518
Age	57	68
Gender	male	male
Size	176 cm	168 cm
Weight	83 kg	82 kg
Smoker	no	no
Drug addiction	none	
Diagnosis	liver metastasis (segment III) of NET (neuroendocrine tumor)	cholangiocellular carcinoma
Therapy	atypical liver resection	hemihepatectomy right
Medication	Somatuline 60 mg (every 28 days)	Metropolol 100 mg ret. (1-0-0,5)<br>Dekristol 20000 IE (every 14 days)<br>Finasterid 5 mg (1-0-0), Propiverin 5 mg (1-0-1)<br>Simvastatin 20 mg (0-0-0-1), Metformin 500 mg (1-0-1)<br>Ramipril 5 mg (1-0-1), Doxazosin 2 mg (0-0-1)<br>HCT 12.5 mg (1-0-0), Madopar 125 mg (1-0,5-1-0,5)<br>Travoprost AT (0-0-0-1), Metamizol 500 mg (1-1-1)
Past medical history	condition after hemikolektomie 2011, liver cyst segment IV	diabetes mellitus, hypertension
Chemotherapy	no	no
HIV 1 + 2	negative	negative
Hepatitis A	negative (immunization protection)	negative (immunization protection)
Hepatitis B	negative	negative
Hepatitis C	negative	negative

Supplementary Table S2. List of metabolites included in the AbsoluteIDQ p180 Kit (Biocrates Life Science AG).

Metabolite class	Metabolites
Amino acids	Ala (Alanine), Arg (Arginine), Asn (Asparagine), Asp (Aspartate), Cit (Citrulline), Glu (Glutamate), Gln (Glutamine), Gly (Glycine), His (Histidine), Ile (Isoleucine), Leu (Leucine), Lys (Lysine), Met (Methionine), Orn (Ornithine), Phe (Phenylalanine), Pro (Proline), Ser (Serine), Thr (Threonine), Trp (Tryptophan), Tyr (Tyrosine), Val (Valine),
Biogenic amines	Ac-Orn (Acetylornithine), alpha-AAA (alpha-Aminoadipic acid), ADMA (Asymmetric dimethylarginine), Carnosine, Creatinine, DOPA (Dihydroxyphenylalanine), Dopamine, Histamine, c4-OH-Pro (cis-4-Hydroxyproline), t4-OH-Pro (trans-4-Hydroxyproline), Kynurenine, Met-SO (Methionine sulfoxide), Nitro-Tyr1

	(Nitrotyrosine), PEA (Phenylethylamine), Putrescine, Sarcosine, Serotonin, Spermidine, Spermine, SDMA (Symmetric dimethylarginine), Taurine,
Monosaccharides	H1 (Hexoses) (including glucose)
Acylcarnitines	C0 (Carnitine), C2 (Acetylcarnitine), C3 (Propionylcarnitine), C3-OH (Hydroxypropionylcarnitine), C3:1 (Propenoylcarnitine), C4 (Butyrylcarnitine), C4-OH (C3-DC) (Hydroxybutyrylcarnitine (Malonylcarnitine)), C4:1 (Butenylcarnitine), C5 (Valerylcarnitine), C5-DC (C6-OH) (Glutarylcarnitine (Hydroxyhexanoylcarnitine)), C5-M-DC (Methylglutarylcarnitine), C5-OH (C3-DC-M) (Hydroxyvalerylcarnitine (Methylmalonylcarnitine)), C5:1 (Tiglylcarnitine), C5:1-DC (Glutaconylcarnitine), C6 (C4:1-DC) (Hexanoylcarnitine (Fumarylcarnitine)), C6:1 (Hexenoylcarnitine), C7-DC (Pimeloylcarnitine), C8 (Octanoylcarnitine), C9 Nonaylcarnitine, C10 (Decanoylcarnitine), C10:1 (Decenoylcarnitine), C10:2 (Decadienylcarnitine), C16:1 (Hexadecenoylcarnitine), C12 (Dodecanoylcarnitine), C12-DC (Dodecanedioylcarnitine), C12:1 (Dodecenoylcarnitine), C14 (Tetradecanoylcarnitine), C18 (Octadecanoylcarnitine), C14:1 (Tetradecenoylcarnitine), C14:1-OH (Hydroxytetradecenoylcarnitine), C14:2 (Tetradecadienylcarnitine), C14:2-OH (Hydroxytetradecadienylcarnitine), C16 (Hexadecanoylcarnitine), C16-OH (Hydroxyhexadecanoylcarnitine), C16:1-OH (Hydroxyhexadecenoylcarnitine), C16:2 (Hexadecadienylcarnitine), C16:2-OH (Hydroxyhexadecadienylcarnitine), C18:1 (Octadecenoylcarnitine), C18:1-OH (Hydroxyoctadecenoylcarnitine), C18:2 (Octadecadienylcarnitine);
Glycerophospholipids	lysoPC a C14:0, lysoPC a C16:0, lysoPC a C16:1, lysoPC a C17:0, lysoPC a C18:0, lysoPC a C18:1, lysoPC a C18:2, lysoPC a C20:3, lysoPC a C20:4, lysoPC a C24:0, lysoPC a C26:0, lysoPC a C26:1, lysoPC a C28:0, lysoPC a C28:1, PC aa C24:0, PC aa C26:0, PC aa C28:1, PC aa C30:0, PC aa C30:2, PC aa C32:0, PC aa C32:1, PC aa C32:2, PC aa C32:3, PC aa C34:1, PC aa C34:2, PC aa C34:3, PC aa C34:4, PC aa C36:0, PC aa C36:1, PC aa C36:2, PC aa C36:3, PC aa C36:4, PC aa C36:5, PC aa C36:6, PC aa C38:0, PC aa C38:1, PC aa

	C38:3, PC aa C38:4, PC aa C38:5, PC aa C38:6, PC aa C40:1, PC aa C40:2, PC aa C40:3, PC aa C40:4, PC aa C40:5, PC aa C40:6, PC aa C42:0, PC aa C42:1, PC aa C42:2, PC aa C42:4, PC aa C42:5, PC aa C42:6, PC ae C30:1, PC ae C30:2, PC ae C32:1, PC ae C32:2, PC ae C34:0, PC ae C34:1, PC ae C34:2, PC ae C34:3, PC ae C36:0, PC ae C36:1, PC ae C36:2, PC ae C36:3, PC ae C36:4, PC ae C36:5, PC ae C38:0, PC ae C38:1, PC ae C38:2, PC ae C38:3, PC ae C38:4, PC ae C38:5, PC ae C38:6, PC ae C40:1, PC ae C40:2, PC ae C40:3, PC ae C40:4, PC ae C40:5, PC ae C40:6, PC ae C42:0, PC ae C42:1, PC ae C42:2, PC ae C42:3, PC ae C42:4, PC ae C42:5, PC ae C44:3, PC ae C44:4, PC ae C44:5, PC ae C44:6
Sphingomyelins	SM (OH) C14:1, SM C16:0, SM C16:1, SM (OH) C16:1, SM C18:0, SM C18:1, SM C20:2, SM (OH) C22:1, SM (OH) C22:2, SM C22:3, SM C24:0, SM C24:1, SM (OH) C24:1, SM C26:0, SM C26:1

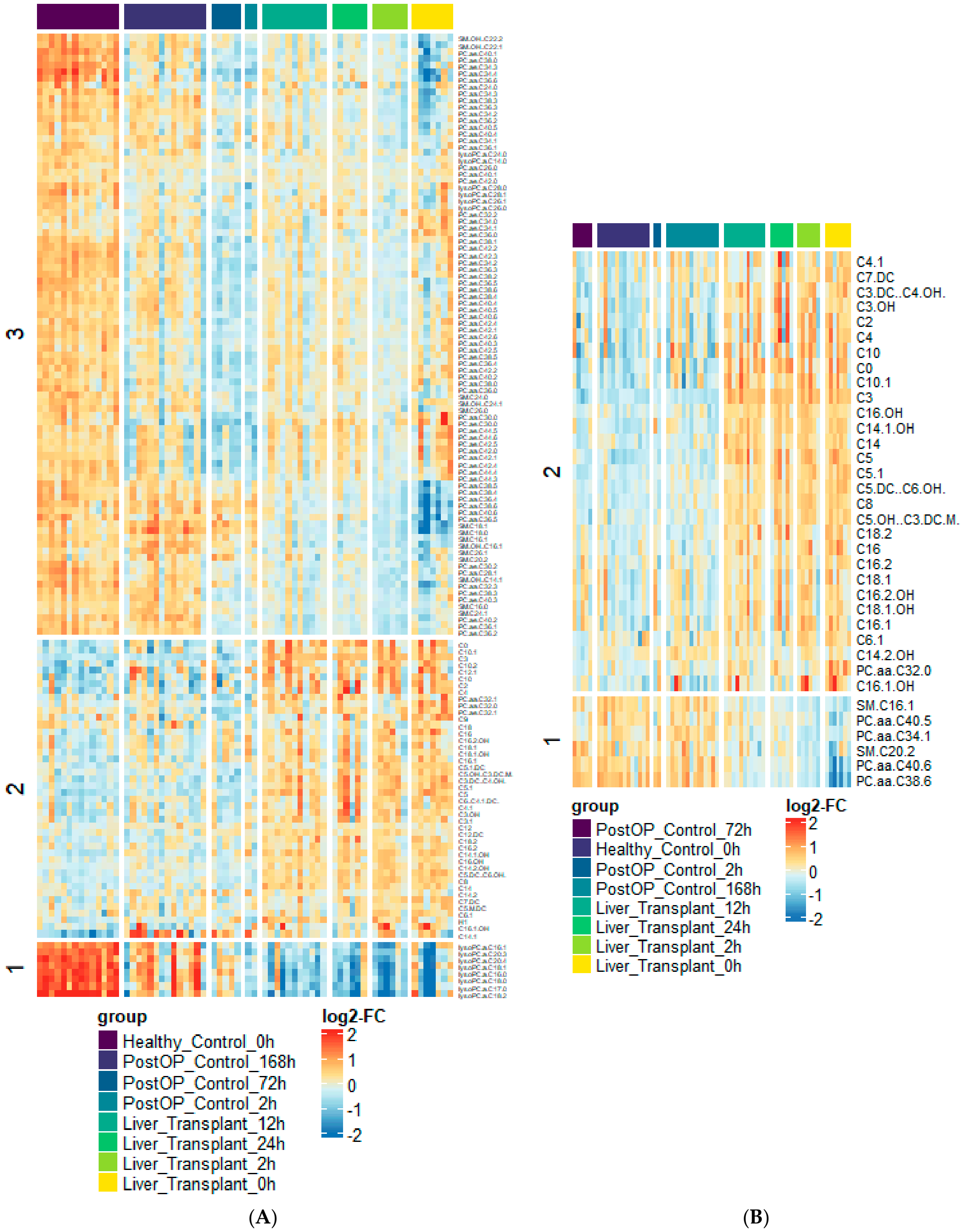

Supplementary Figure S1. Metabolites changed in patients with an acute-on-chronic liver failure before and after liver transplantation and identified metabolic biomarker set for hepatocellular injury (**A**) Log2 fold changes of metabolite concentrations in patients with an acute-on-chronic liver failure before and at several time points after liver transplantation. (**B**) Detailed view of metabolites found with significant log2 fold changed concentrations in patients with hepatocellular injury compared to post OP and healthy control patients potentially serving as translational metabolic biomarker set for hepatocellular injury. Relevant metabolites were identified as stated in the materials and methods section.

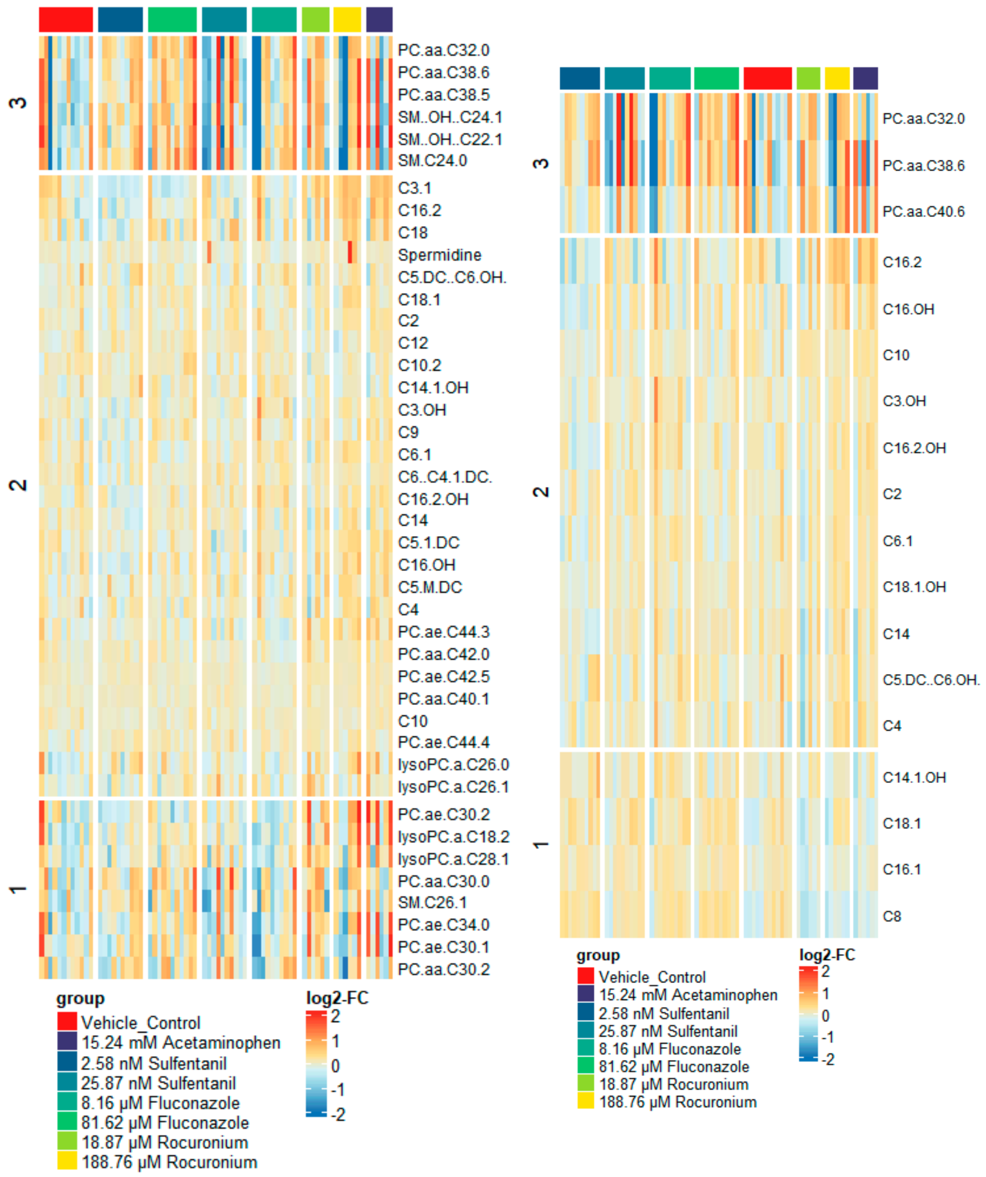

(**A**) (**B**)

Supplementary Figure S2. Detailed view of all metabolites changed in each PHH sample in response to the exposure with several drugs. (**A**) log2 fold changes of metabolite concentrations in primary human hepatocytes from two donors in comparison to vehicle control. (**B**) Detailed view of log2 fold changes of selected metabolites found with significant log2 fold changed concentrations in PHH from two donors opposed to log2 fold changes.

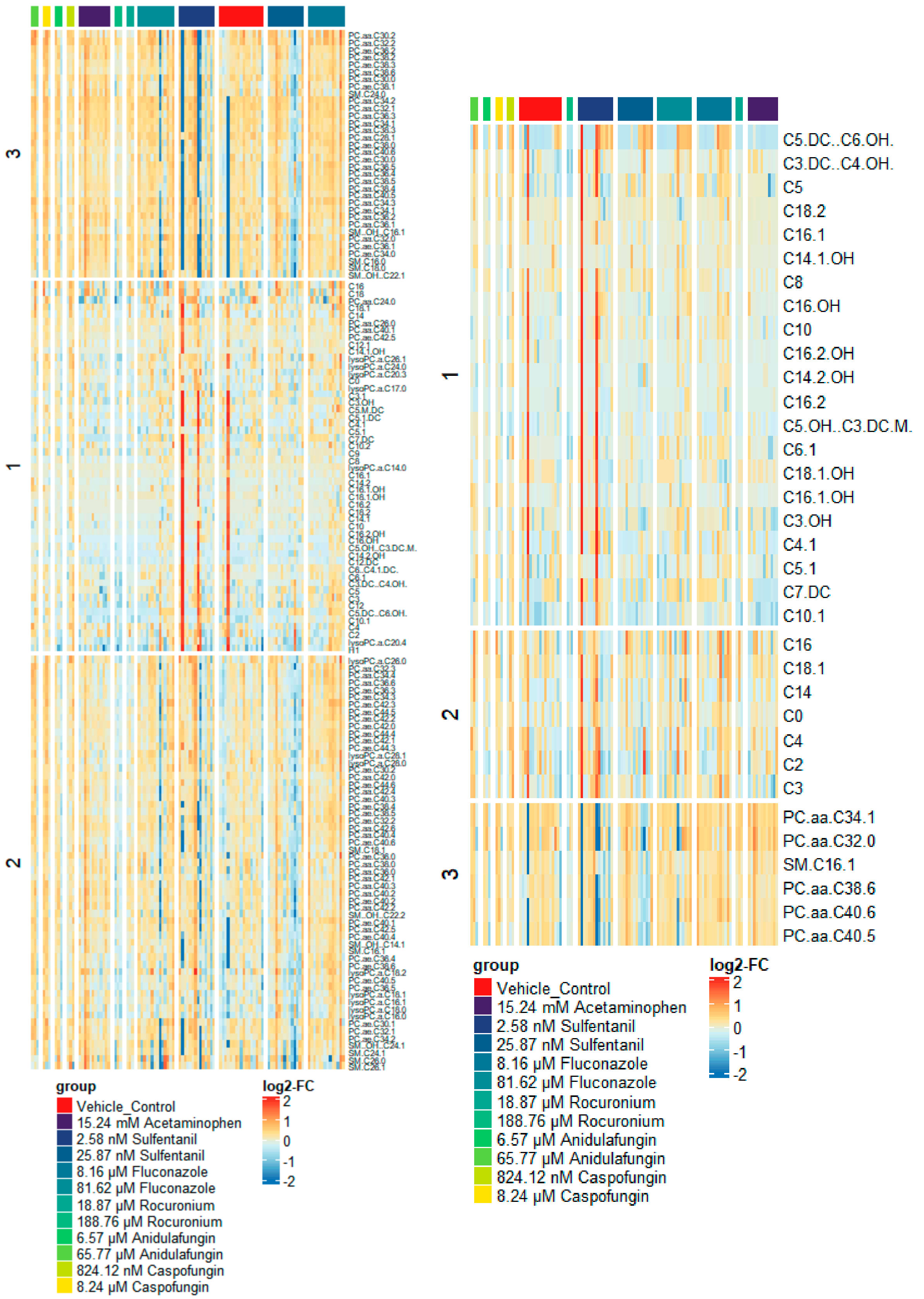

(A) (B)

Supplementary Figure S3 Detailed view of all metabolites changed in each HepG2/C3A hepatocytes sample in response to the exposure with several drugs (**A**) log2 fold changes of metabolite concentrations HepG2/C3A in comparison to vehicle control (for a detailed heatmap of log2 fold changes for each sample (**B**) Detailed view of log2 fold changes of selected metabolites found with significant log2 fold changed concentrations in HepG2/C3A in comparison to vehicle control.
